# An interprofessional approach to treat bodily distress disorder in Swiss primary care – a quality improvement study

**DOI:** 10.1080/13814788.2025.2599579

**Published:** 2026-01-08

**Authors:** Stefania Di Gangi, Julia Hennemann, Emanuel Brunner, Oliver Senn, Stefan Büchi

**Affiliations:** ^a^ Institute of Primary Care Zurich, University of Zurich and University Hospital Zurich, Zurich, Switzerland; ^b^ mediX General Practice Group Zurich, Zurich, Switzerland; ^c^ Department of Health, OST – Eastern Swiss University of Applied Sciences, St. Gallen, Switzerland

**Keywords:** Bodily distress disorder, PHQ-15, general practice, physiotherapy, physical activity

## Abstract

**Background:**

Managing bodily distress disorder (BDD) requires an interprofessional, holistic therapeutic approach, which can be challenging to implement in routine care.

**Objectives:**

The aim was to evaluate a care pathway for patients with BDD involving general practitioners (GPs) and physiotherapists.

**Methods:**

Participants were patients aged 18 years or older with BDD symptoms and the PHQ-15 (Patient Health Questionnaire 15-Item) score > 9. The treatment consisted of patient education by GPs about BDD and stress, sleep interventions, physical exercises and support to develop an active lifestyle (> 150 min per week of moderate-intensity physical activity), as measured with the Physical Activity Vital Sign (PAVS). Patient outcomes (PHQ-15 and PAVS) were compared at baseline and after six months. A survey assessed the perspectives of both patients and health professionals about the treatment.

**Results:**

A total of 70 patients treated by 11 GPs and 6 physiotherapists were involved. Comparing outcomes at baseline vs. after six months (median [IQR]): PHQ-15 decreased, 14 [11, 17] vs. 8 [5, 12], *p* < 0.001; PAVS increased, 60 [30, 120] vs. 120 [60, 180], *p* < 0.001; GP confidence (scale 0–100) in treating BDD increased, 50 [38, 66] vs. 82 [66, 85], *p* = 0.005. Feedback from patients, GPs and physiotherapists about the intervention was positive.

**Conclusion:**

The interprofessional care pathway for patients with BDD had a positive impact on patient outcomes, GP treatment confidence, and was well-received by both patients and health professionals. It can be adapted across primary care systems and tailored to local contexts to improve the quality of care.

## KEY MESSAGES

- Educating patients about bodily distress disorder (BDD) and stress, sleep hygiene and supporting an active lifestyle improves patient outcomes.
- GPs’ treatment confidence improves with collaboration with physiotherapists.
- The interdisciplinary approach to BDD can be adapted across primary care systems and tailored to local contexts to improve the quality of care.

## Introduction

Persistent physical symptoms, i.e. functional and somatoform bodily complaints, are among the most common reasons for consultation in primary care and, irrespective of the cause, they are an increasing global burden in terms of years lived with disability and disability-adjusted life years as well as high health-care costs [1]. About one-third of patients in primary care have persistent physical symptoms that cannot be attributed to a recognised disease [1,2].

Bodily distress disorder (BDD) is a new diagnostic concept, defined by the ICD (International Classification of Diseases)-11 [3]. It is characterised by persistent physical symptoms and obsessive focus on them. This focus interferes with daily functioning and an appropriate clinical examination, investigation, or reassurance does not alleviate it. The new term, “bodily distress disorder,” replaces the old somatoform disorder category used in the ICD-10. However, the new definition does not require that the symptoms be “medically unexplained”; they could be related to an underlying disease. Although the new definition supports a more holistic approach, it has led to confusion regarding terminology [4]. Moreover, with the new definition, little is known about the prevalence of BDD, and the implemented treatment strategies [5]. A holistic patient-centred approach, based on the individual’s specific symptoms and needs, is recommended [5,6]. Swiss and German-speaking clinical guidelines recommend a biopsychosocial approach, emphasising early recognition, interdisciplinary collaboration, and multimodal treatment including psychotherapy, physiotherapy and lifestyle interventions [7]. General practitioners (GPs) should play the role of both the initial point of contact and the coordinator of appropriate diagnostic and treatment procedures. However, implementation remains inconsistent, and many patients do not receive care aligned with these guidelines [8]. Many patients visit various medical specialists to find the physical cause of their symptoms. This often leads to a series of somatic diagnostic procedures that are not well-coordinated, resulting in inappropriate treatment with minimal integration between physical and mental health services [8,9]. On the other hand, there is a lack of specific studies, in particular in Switzerland, evaluating the integrated care models for BDD.

In light of this, the project aimed to evaluate a care pathway for BDD involving GPs and physiotherapists. It was a quality assurance initiative in a Swiss primary care setting. It aimed to evaluate if the care model would reduce symptoms and improve health behaviours in affected patients, and if it would enhance the diagnostic and therapeutic competence of participating health professionals.

## Methods

### Design

This quality improvement project was conducted in a large general group practice in Zurich, Switzerland, with more than 60 staff, including GPs, specialists and consultants. The practice offers guideline-oriented, evidence-based medicine with a coordinated team and an on-site institute for physiotherapy. All GPs and physiotherapists were trained on BDD and stress, and how to use patient materials. The definition of BDD was based on the 6C2” Disorders of bodily distress or bodily experience” in ICD-11^3^ (Supplementary Material1 Box 1).

**Figure 1.**
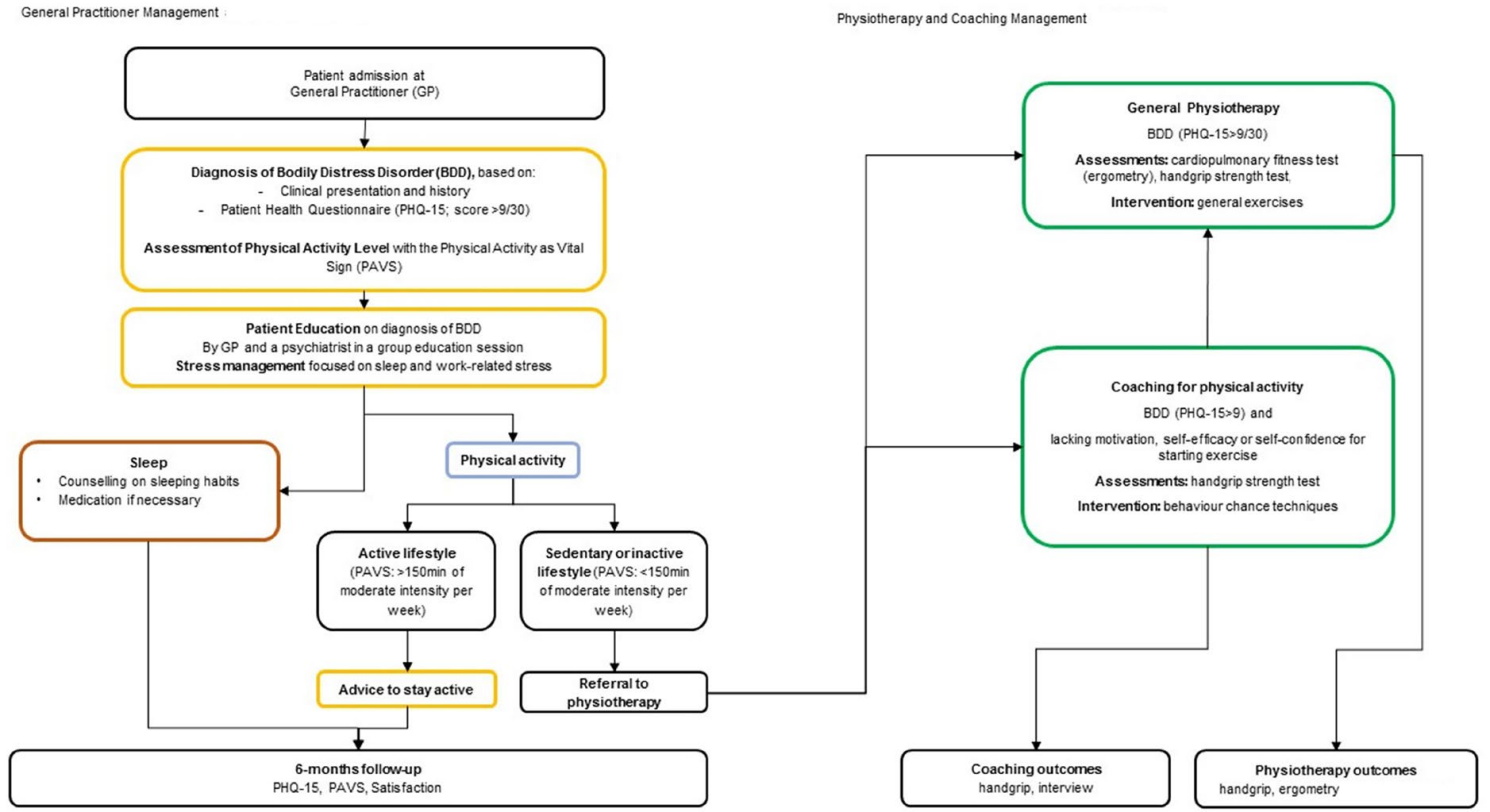
Treatment pathway for patients with bodily distress disorder: 1st April–31st October 2023. Follow-up: 31st March 2024. Notes: PAVS: Physical Activity Vital Sign; active lifestyle: meeting the recommended level of physical activity; sedentary or inactive lifestyle: not meeting the recommended level of physical activity.

Patients aged 18+ with 3+ months of persistent BDD symptoms who saw a GP between 1 April 2023 and 31 October 2023 were assessed using the 15-item Patient Health Questionnaire (PHQ-15) [10] German version. Patients with a PHQ-15 severity score of 9+ (max 30), indicating moderate to severe intensity [11] were considered (Figure 1).

### Treatment pathway

During the initial consultation, GPs educated patients using printed materials about BDD, stress, and impaired well-being (Supplementary Material 2). If patients reported issues with sleeping, they were educated on healthy sleeping habits and given medication, if necessary. All patients were invited to attend a 75-minute group education session led by a psychiatrist working as a specialist in the group practice. These sessions were designed for three to six people to discuss their symptoms and general BDD-related issues.

Patient-reported physical activity was assessed using Physical Activity Vital Sign (PAVS) [12]. Patients meeting the recommended level of moderate-intensity physical activity (>150 min per week) [13] were advised to stay active. The others were referred to physiotherapy for general physical exercises, including endurance and strength training.

Initially, physical exercises were done in one-to-one sessions. Later, patients moved to group training, supervised by physiotherapists. Two to three sessions were planned per week for three months.

Patients lacking motivation, self-efficacy or self-confidence for starting physical exercises were referred to specialised physiotherapy (coaching for physical activity) focusing on enhancing patients’ motivation for behaviour change, helping them plan and organise physical activity into daily life. Patients could transition to regular physiotherapy if they and the physiotherapist agreed.

Follow-ups were done by GPs 6 months after inclusion. Patients were also invited to complete a pen-and-paper survey about their experience and satisfaction. The questionnaires were sent to the practice, and the data were recorded electronically.

### Patient data and outcomes

Demographic data (age in years and sex) were collected for all patients. To screen for anxiety and depression, the Hospital Anxiety and Depression Scale (HADS) questionnaire (divided into an anxiety subscale (HADS-A) and a depression subscale (HADS-D) [14] was used at baseline. In addition, information about issues with sleeping (included in the PHQ-15 questionnaire) was stored as a baseline variable.

The primary outcome was the difference in PHQ-15 scores at the six-month follow-up (post-treatment) compared to the baseline evaluation (pre-treatment). PHQ-15 was considered as both a continuous or categorical score (0–4: minimal, 6–9: low, 10–14: medium, 15–30: high burden) [11]. The outcome subgroups were physiotherapy and group education sessions. Secondary outcomes were: 1) pre-post PAVS, and physical fitness assessments, including ergometry for testing cardiorespiratory fitness (maximal performance in Watt) and handgrip for muscular strength (maximal strength in kg); 2) patient-reported follow-up assessment of relative change in symptom burden (from −100% to +100%), perceptions and experiences (open-ended questions), and satisfaction with treatment, measured with numeric rating scales 0–10 (Supplementary Material 2).

We also identified factors associated with the primary outcome.

### Professional experience by GPs and physiotherapists

A pre-post online survey in German was disseminated to GPs and physiotherapists involved in the treatment. The following data were collected: demographics (age, sex), work experience, workload, views on BDD and treatment options (yes/no or single choice questions), confidence in diagnosing and treating BDD patients (rating scale 0–100), treatment experience and interprofessional collaboration (questions measured on rating scales 0–100 or 0–10) (Supplementary Material 2).

### Statistical analysis

All analyses were carried out using the statistical package R version 4.4.3 [15]. A *p* ≤ 0.05 was used to determine statistical significance.

Data were described as numbers and percentages, n(%), for categorical or binary variables and as mean (standard deviation (SD)) or median [interquartile range (IQR)], as appropriate, for continuous variables. An available case analysis was performed and the number of non-missing observations was reported. For group comparisons between the two time points (pre/post) and between subgroups (physiotherapy yes/no; group education session yes/no) chi-squared test was used for categorical or binary variables and the t-test or the Wilcoxon test, as appropriate, for continuous variables. Differences post-pre were also reported with a 95% confidence interval (CI).

Linear regression analysis, univariable and multivariable, was performed to identify factors associated with the difference in PHQ-15 scores (post – pre). Regression results were reported as estimates with 95% CI.

Qualitative analysis of the open-ended questions in the patient survey was performed using a framework analysis [16]. Tags, assigned by the researcher from patient texts, were used as conceptual codes to describe experience, needs or suggestions for improvement. A specification of all tags is provided (Supplemental Material 1 Table 1). Tags were visualised using word cloud figures, where more frequent tags appeared larger than the less frequent ones [17].

## Results

A total of 70 patients were included. Baseline characteristics were reported in Table 1.

**Table 1.** Baseline characteristics of patients.

	*N* = 70
Age (mean (SD))	39.91 (9.78) (min = 19, max = 63)
Sex = female N(%)	57 (81.4)
Sleep issue = Yes N(%)	66 (94.3)
HADS A (median [IQR])	12 [8, 15] (min = 0, max = 21)
	N(%)
Normal (0–7)	12 (17.1)
Borderline (8–10)	13 (18.6)
Abnormal (11–21)	45 (64.3)
HADS D (median [IQR])	8 [4, 11.75] (min = 0, max = 17)
	N(%)
Normal (0–7)	31 (44.3)
Borderline (8–10)	14 (20.0)
Abnormal (11–21)	25 (35.7)
Meeting the recommended level of physical activity (active lifestyle) N(%)	Missing = 7
Yes (PAVS ≥ 150 min/week)	9 (14.3)
No (PAVS < 150 min/week)	54 (85.7)

Abbreviations. Min: Minimum; max: Maximum; HADS: Hospital anxiety and depression scale questionnaire; HADS A: Anxiety subscale from HADS questionnaire; HADS D: Depression subscale from HADS questionnaire; PAVS: Physical activity vital sign.

Patients were aged 39.91 (9.78) (mean (SD)) years, and the majority, 57 (81.4%), were female. Almost all patients 66 (94.3%) reported sleeping problems. Most patients reported an abnormal anxiety score, 45 (64.3%), and did not meet the recommended physical activity level, 54 (85.7%).

The majority of patients, 46 (65.7%), received physiotherapy. Others received coaching for physical activity, 6 (8.6%), or advice to stay active 14 (20%). Group education session was received by 34 (48.6%) patients, and 25 of these patients also received physiotherapy.

### Patient outcomes and survey

**Figure 2.**
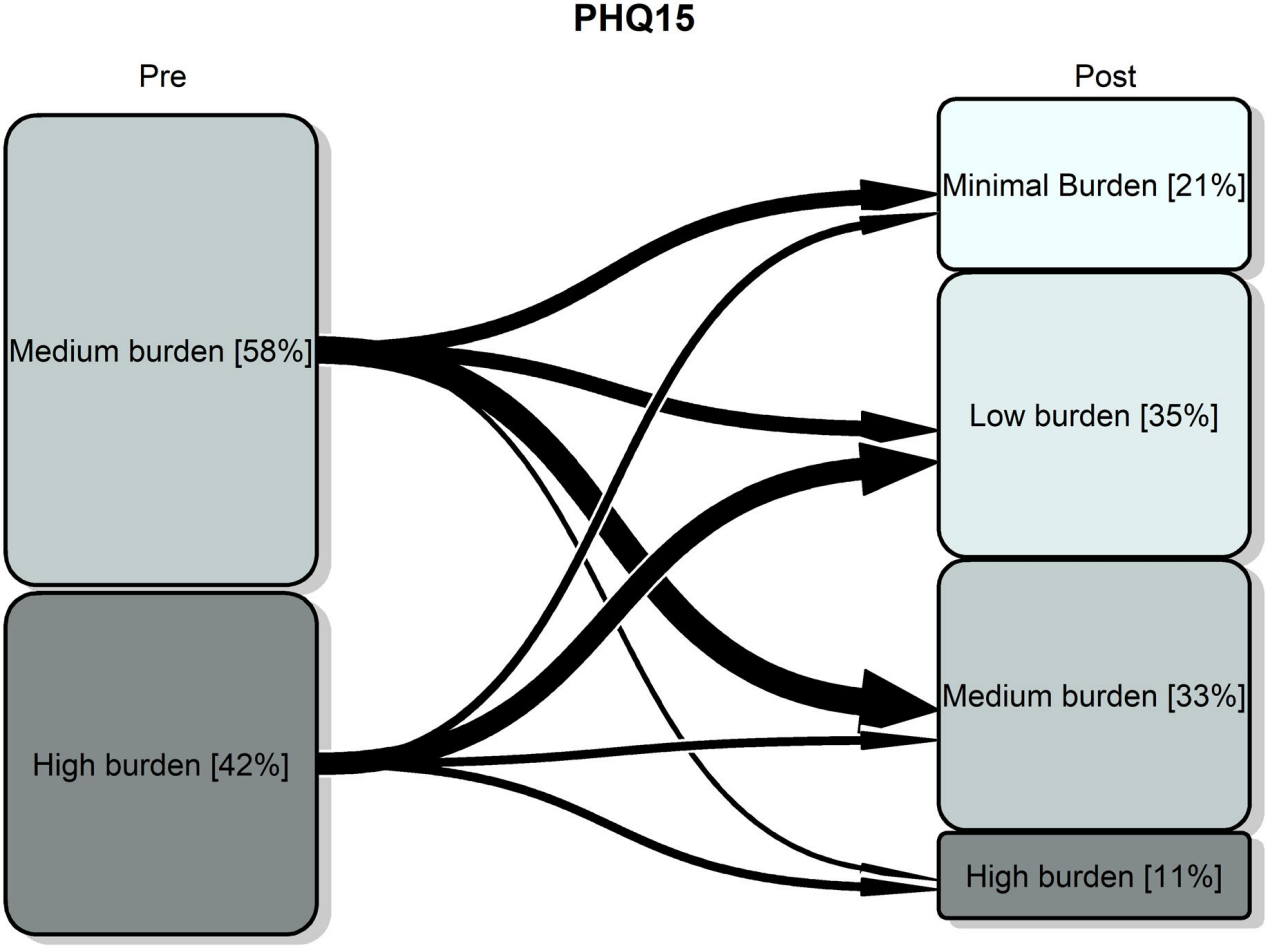
Transition plot of severity category based on PHQ-15 score at baseline (pre) and at 6-month follow-up (post). Notes: Minimal burden: PHQ-15: 0–4; low burden: 6–9; medium burden: 10–14; high burden: 15–30. Percentages of patients in each group were reported.

Outcomes pre-post were reported in Table 2. A total of 57 patients were followed up. PHQ-15 score reduced from 14 [11, 17], median[IQR], at baseline to 8 [5, 12] at follow-up, *p* < 0.001, difference (95% (CI)):- 6 (−8, −3). Similar results were found in treatment subgroups: physiotherapy and group education sessions. The proportion of patients with a high burden of symptoms reduced from 42% at baseline to 11% at follow-up (Figure 2). The majority of patients, 36 (63.2%) improved; only 2 (3.5%) patients worsened from medium burden to high burden of symptoms and 19 (33.3%) remained stable. The multivariable regression analysis (Table 3) evidenced that the improvement post-pre in PHQ-15 increased with increasing pre-anxiety score (estimate 95%(CI): −0.39 (−0.70; −0.07)) and it was greater in patients who received group therapy than in those who did not, −2.90 (−5.63; −0.16).

**Table 2.** Evaluation of patient outcomes.

	Pre-treatment (baseline)	Post-treatment (6-month follow-up)	Difference (Post-Pre) (95% CI)*	*p*-value**
**PHQ-15**				
Overall mean (SD)	14.39 (3.50)	8.82 (4.82) *N* = 57		
median [IQR] (min, max)	14 [11, 17] (min = 10, max = 24)	8 [5, 12] (min = 1, max = 22)	− 6 (−8, −3)	<0.001
Subgroup analysis median [IQR]				
Physiotherapy Yes *N* = 46 No *N* = 24	14 [11.25, 16.75] 13.5 [11, 17]	8.5 [5, 11] *N* = 38 8 [5, 14] *N* = 19	− 5.5 (−8, −3.5) − 5.5 (−9, −0.5)	<0.001 0.009
Group education session Yes *N* = 34 No *N* = 36	14 [12.25, 16.75] 13.5 [11, 17]	8 [4, 10] *N* = 29 10 [6, 14] *N* = 28	−6 (−10, −4) −3.5(−7.5, −0.5)	<0.001 0.001
**PAVS** minutes/week mean (SD)	76.03 (70.10) *N* = 63	134.29 (98.67) *N* = 56		
median [IQR] (min, max) Not meeting the recommended level of physical activity N(%)	60 [30, 120] (min = 0, max = 360) 54 (85.7)	120 [60, 180] (min = 0, max = 480) 36 (64.3)	60 (30, 80) −21.4% (−38.3, −4.5)%	<0.001 0.012
**Assessments of physical fitness**	Begin of physiotherapy	End of physiotherapy		
Ergometry maximal performance median [IQR]	162.50 [146.25, 188.75] *N* = 34	165 [156.25, 201.25] *N* = 10		0.423
Handgrip maximal strength kg right median [IQR]	27.33 [24.5, 32] *N* = 36	31.16 [25.25, 32.74] *N* = 12		0.294
left median [IQR]	25 [22.33, 29.16] *N* = 36	28.50 [25.33, 30.66] *N* = 12		0.137

* Estimated using the bias-corrected and accelerated bootstrap method with 1000 resamples.

** *p*-values from Wilcoxon or chi-square test were reported.

Abbreviations. PHQ-15: Patient health questionnaire 15-item somatic symptom severity scale; min: Minimum; max: Maximum; CI: Confidence interval; PAVS: Physical activity vital sign.

**Table 3.** Linear regression analysis of the difference in PHQ-15 score post – pre (post: 6-month follow-up; pre: baseline).

	Univariable Estimate (95% CI); *p*-value	Multivariable* (57 patients) Estimate (95% CI); *p*-value
Age	−0.01 (−0.18; 0.15); *p* = 0.866	
Sex (Female)	−0.81 (−4.77; 3.15); *p* = 0.683	
Sleep Issue (pre)	−0.93 (−6.83; 4.96); *p* = 0.752	
Difference in exercise (PAVS) post-pre minutes/week	−0.01 (−0.03; 0.00); *p* = 0.164	
HADS A (pre)	−0.48 (−0.77; −0.18); *p* = 0.002	−0.39 (−0.70; −0.07); *p* = 0.016
HADS D (pre)	−0.35 (−0.68; −0.02); *p* = 0.040	−0.25 (−0.59; 0.08); *p* = 0.132
Group education session	−2.48 (−5.42; 0.46); *p* = 0.097	−2.90 (−5.63; −0.16); *p* = 0.038
Physiotherapy	−1.74 (−4.90; 1.43); *p* = 0.276	

* The selection of variables for the multivariable model was based on a stepwise backward approach, starting with a full model including all variables and excluding them using the Akaike information criterion (AIC).

Abbreviations. PHQ-15: Patient health questionnaire 15-item somatic symptom severity scale; CI: Confidence interval; HADS: Hospital anxiety and depression scale questionnaire; HADS A: Anxiety subscale from HADS questionnaire; HADS D: Depression subscale from HADS questionnaire; PAVS: Physical activity vital sign.

Physical activity levels increased after six months, from a median [IQR] of 60 [30, 120] to 120 [60, 180] minutes per week, *p* < 0.001, with a reduction of 21.4% (95% CI: 4.5%-38.3%) in the proportion of patients not meeting the recommended level (Table 2). No significant differences were found in physical fitness assessments.

Patients highly recommended the treatment (median [IQR] 9 [7, 10] over a scale of 0–10) and were satisfied with the interprofessional management, 8 [4, 10], patient material, 9 [7, 10], and physiotherapy, 8 [6, 10] (Table 4). Physical exercises, skills and specific techniques, and establishing a routine were the most common factors that patients thought were relevant to improving their wellbeing (Supplementary Material 1 Figure 1 and Supplementary Material 1 Table 1). Acquiring self-efficacy, practicing skills, physical exercise, and understanding their symptoms or treatment indications were key moments in patients’ progress (Supplementary Material 1 Figure 2) and were also suggested as aspects that would have helped them better manage physical stress symptoms in the future (Supplementary Material 1 Figure 3). What patients found critical about the treatment and in need of more support was the psychology, the time management, the patient education and the support (Supplementary Material 1 Figure 4). In fact, some patients reported the need for “more time for patients” or that “the time for physiotherapy was limited” or that “the routine need time and patience” (Supplementary Material 1 Table 1).

**Table 4.** Survey of patients with bodily distress disorder at 6-month follow-up.

	Number of Patients	57
Age (mean (SD))		40.90 (9.15)
Sex n (%)	Male	10 (17.5)
	Female	47 (82.5)
Could you explain today what bodily distress disorder is to someone you know in an understandable way? n (%)	Yes	50 (92.6)
	No	4 (7.4) *N* = 54
If you had to assign your physical symptoms to different causes, what percentage would you assign to the following aspects		
Psyche (mean (SD)) *N* = 55		36.17 (19.85)
Body (mean (SD)) *N* = 55		22.90 (16.59)
Stress (mean (SD)) *N* = 55		44.90 (21.26)
How has the impairment caused by the bodily distress disorder symptoms changed in the last 6 months? n (%)	Reduction of symptoms	42 (79.2)
	Unchanged	6 (11.3)
	Increase of symptoms	5 (9.4) *N* = 53
% of change (%) (details of question above)	−100	1 (1.9)
	−80	9 (17.0)
	−70	4 (7.5)
	−60	10 (18.9)
	−50	1 (1.9)
	−40	8(15.1)
	−30	1 (1.9)
	−20	8 (15.1)
	0	6 (11.3)
	20	2 (3.8)
	30	1 (1.9)
	40	1 (1.9)
	60	1 (1.9)
Do you believe that the project will enable you to cope better with physical stress symptoms in the future n (%)	Yes	45 (91.8)
	No	4 (8.2) *N* = 49
The medical discussion with the explanations and illustrative material was helpful scale 0 –10 (median [IQR])		9 [7, 10] *N* = 53
I found the discussions with the physiotherapist helpful scale 0–10 (median [IQR])		8 [7, 10] *N* = 41
I found the training with physiotherapy helpful scale 0–10 (median [IQR])		8 [6, 10] *N* = 39
I noticed that the doctor and physiotherapist worked together as a team to manage my treatment scale 0–10 (median [IQR])		8 [4, 10] *N* = 43
Would you recommend participation in such a project to friends and acquaintances? scale 0–10 (median [IQR])		9 [7, 10] *N* = 50

Abbreviations. SD: Standard deviation; IQR: Interquartile range.

### Interprofessional experience

**Figure 3.**
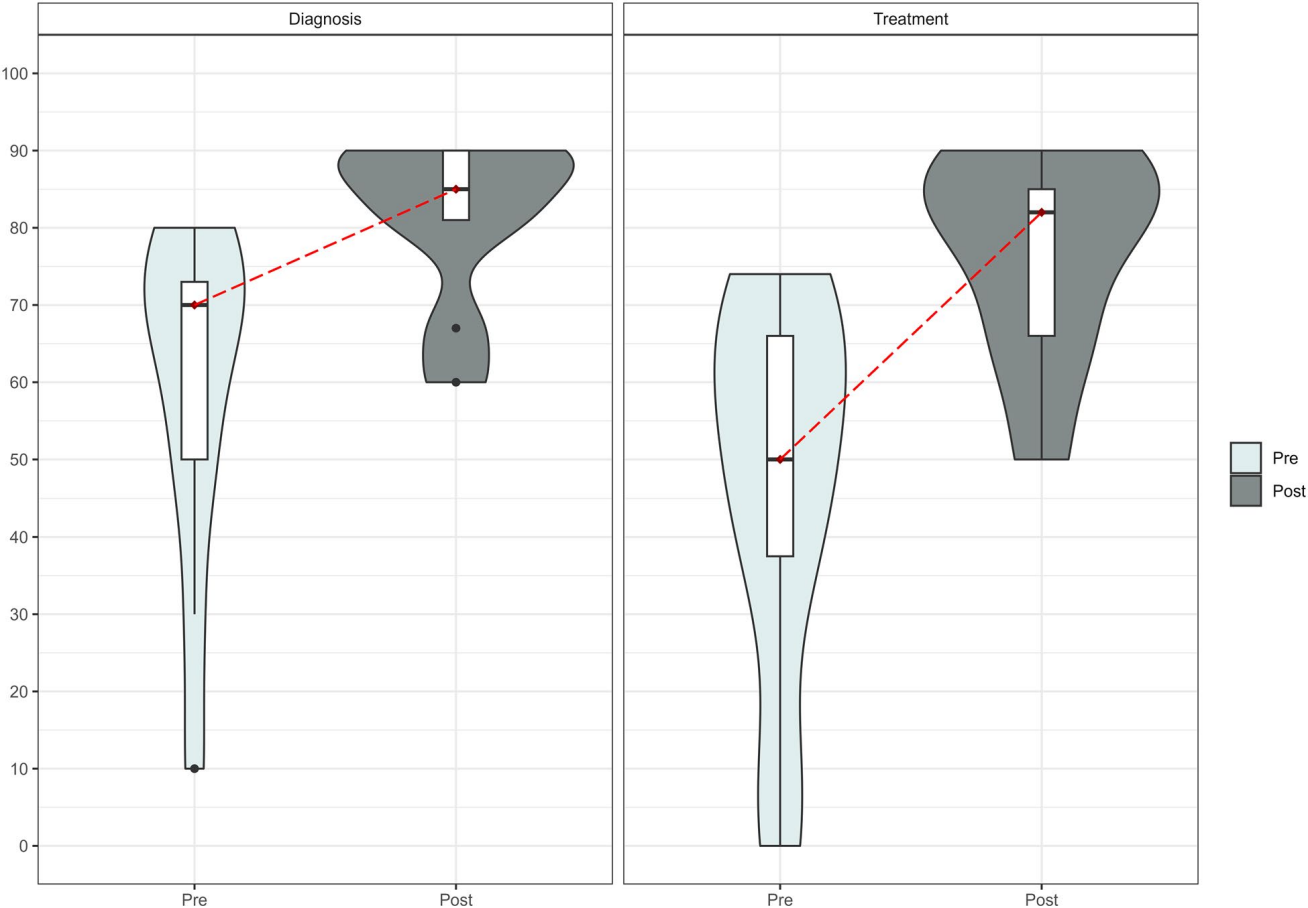
Violin plot of GPs’ confidence in diagnosis and treatment of bodily distress disorder at baseline (pre) and at follow-up (post). Notes: The boxes showed the 75th percentile (the upper horizontal line), median (the black bold horizontal line), and the 25th percentile (the lower horizontal line) of the distribution. The upper whiskers showed the highest value of the variable, which was 1.5 times the interquartile range above the 75th percentile. The lower whiskers indicated the corresponding distance to the 25th percentile value. A rotated kernel density plot, similar to a histogram with infinitely small bin sizes, surrounded the boxes (shaded area) on each side. The pre-post changes were indicated by the dotted red line.

Eleven GPs participated (Supplementary Material 1 Table 2). At follow-up, GPs felt more confident with the diagnosis (median [IQR] post vs. pre: 85 [81, 90] vs. 70 [50, 73], *p* = 0.006, on a scale of 0–100) and the treatment (82 [66, 85] vs. 50 [38, 66], *p* = 0.005) of BDD (Figure 3). GPs had a good experience of working with physiotherapists (8 [8, 9] on a scale of 0–10) and highly recommended the use of the interprofessional approach to other GPs and would continue to use it (10 [9, 10] on a scale of 0–10). More self-confidence (78%), better knowledge of diagnostics (67%) and a clear algorithm (56%) were relevant to GPs when dealing with BDD patients. GPs also need communication training (56%).

Six physiotherapists participated. At follow-up, three physiotherapists reported an increased confidence in dealing with BDD patients, and two out of three reported increased cooperation with GPs after treatment (Supplementary Material 1 Table 3).

## Discussion

### Main findings

A care pathway for patients with BDD involving GPs and physiotherapists was evaluated. After six months, the severity of symptoms (PHQ-15 score) reduced and the majority of patients improved; a higher baseline anxiety score and participation in the group education session were associated with a greater score reduction; physical activity levels increased. Patients and GPs highly recommended the pathway and were satisfied with the interprofessional management. GPs and physiotherapists’ confidence and cooperation in treating BDD increased.

### Comparison with the literature

Regarding diagnosis, we used the recent ICD-11 definition of BDD, which is more inclusive than the previous concept of somatoform disorder [18]. However, this definition is rarely used, and the neutral term still referred to in the literature is “persistent physical symptoms” [1]. This may limit the validity of comparisons across studies. In particular, we found that over 80% of BDD patients are female. Research is needed to establish the sex distribution among BDD patients identified through ICD-11 criteria.

In terms of treatment approach, few studies [19–21] involved GPs and physiotherapists like ours. Others involved GPs and psychotherapists or mental health clinics [8,22,23]. Our findings are consistent with studies demonstrating that a collaborative, interprofessional approach with patients experiencing persistent physical symptoms results in improved patient outcomes [1,23,24], and was highly appreciated by the majority of participants [8,19–21] and by GPs [25]. A higher baseline anxiety score and participation in group education sessions were associated with greater symptom severity reduction. Further investigation is needed, but this finding supports the use of more targeted psychological therapy and highlights the importance of educational groups in psychological therapy.

Our study also provided insights on patients’ perspectives evidenced in the literature [26]: self-management, understanding own symptoms, and receiving treatment indications by GPs are key moments in patients’ progress; patients need education, support -- particularly regarding self-management or referrals to psychotherapists or psychiatrists, and more time for consultation and psychotherapy aid. Patients reported that physical exercise, skills, and routines improved their well-being. This is similar to the body-mind connection linked to body-oriented exercises [27].

The interprofessional approach boosted GPs’ and physiotherapists’ confidence in diagnosing and treating BDD, and improved collaboration. Training was also identified as needed. These results confirmed previous findings [26,27].

### Strengths and limitations

To our knowledge, this is the first evaluation of a care pathway for patients with BDD involving GPs and physiotherapists in Switzerland. The strengths are its consideration of the new BDD ICD-11 definition, its use of validated methods to measure patient outcomes, and its provision of insights into patients’ and healthcare professionals’ perspectives.

The main limitation is the nature of this project. It was conceived to suggest the feasibility of the care pathway and its practice improvement rather than generalisable knowledge. We aimed to bring the treatment up to the current standard of care, but not to determine the efficacy of this treatment and define a new standard of care. Therefore, our findings are location-specific and may not be generalisable. However, they deserve to be shared to support feasible ways to implement evidence-based changes that can improve BDD patients and the health care system. Another limitation is that the treatment pathway was stepped and tailored according to clinical judgement. Although this was intended to be the most beneficial for the patient, the result was a heterogeneous sample comprising diverse clinical scenarios, characteristics and allocations. Additionally, we did not evaluate health service utilisation and its corresponding costs. Moreover, we did not collect patient characteristics that may be predisposing factors for BDD, like education level, sociocultural factors, body-mass index, comorbidities [1], confounders or associated with the physical activity, stress or sleep disturbances (e.g. medications, smoking, alcohol consumption). There were missing values and no information about patient adherence, which could have impacted the physical fitness assessments. Our follow-up was six months, and we could not determine if the reduction in symptoms lost significance at 12 months [21,23]. Finally, the dropout rate due to a lack of attendance by patients could have affected our findings.

### Conclusion and implications for practice

A care pathway for patients with BDD was evaluated. The treatment consisted of patient education by GPs, a group session with a psychiatrist and support by GPs and physiotherapists to develop an active lifestyle. This interprofessional approach aligns with international and national guidelines [7,28]. Our interprofessional care pathway for patients with BDD had a positive impact on patient outcomes, GPs’ treatment confidence, and was well received by both patients and health professionals. However, there is room for improvement. Patients expect further education about their condition and psychotherapeutic support. Therefore, GPs need to improve their communication skills. A good doctor–patient relationship and communication, especially regarding symptom causes, and interprofessional collaboration are key for delivering and succeeding with interventions for BDD. Our approach went in the right direction, though further research is needed as BDD is a new diagnostic concept, especially for GPs.

Adopting this approach as routine care for BDD in Switzerland is possible, given that the country’s healthcare system allows for an integrated model of care that includes primary care physiotherapy centres and psychotherapy in cooperation with GPs. Evidence supports the interdisciplinary approach involving physiotherapists [29], but this may not be feasible in European countries like Italy, where GPs are isolated professionals. In those countries, it is suggested to strengthen the role of general practitioners and offer them specialised training on BDD management [30]. This should include establishing trust with patients, providing education to improve their well-being, and making their healthcare journey shorter. Mobile health tools could be used for education and monitoring. In conclusion, the evaluated care pathway can be scaled and adapted across diverse primary care systems by leveraging existing infrastructure, community resources, and digital innovations. Tailoring the model to local contexts ensures its sustainability and improves patient outcomes.

## Supplementary Material

Supplemental Material

## Data Availability

The datasets used and/or analysed during the project are available from the corresponding author on reasonable request.
